# The involvement of Elf5 in regulating keratinocyte proliferation and differentiation processes in skin

**DOI:** 10.1371/journal.pone.0316134

**Published:** 2025-01-03

**Authors:** Anhua Hu, Maximilian E. Pickup, Maryam A. Lawal, Hetal J. Patel, Mohammed I. Ahmed

**Affiliations:** School of Science and Technology, Nottingham Trent University, Nottingham, United Kingdom; Northwestern University, UNITED STATES OF AMERICA

## Abstract

Skin and hair development is regulated by multitude of programs of activation and silencing of gene expression to maintain normal skin and hair follicle (HF) development, homeostasis, and cycling. Here, we have identified E74-like factor 5 (Elf5) transcription factor, as a novel regulator of keratinocyte proliferation and differentiation processes in skin. Expression analysis has revealed that Elf5 expression was localised and elevated in stem/progenitor cell populations of both the epidermis (basal and suprabasal) and in HF bulge and hair germ stem cell (SCs) compartments during skin and hair development and cycling. Expressional and functional analysis using RT-qPCR, western blot and colony forming assays, revealed that Elf5 plays an important role in regulating keratinocyte proliferation and differentiation processes as well as potentially determining cell fate by regulating the stem/progenitor cell populations in skin and HFs. These data will provide a platform for pharmacological manipulation of Elf5 in skin, leading to advancements in many areas of research, including stem cell, regenerative medicine, and ageing.

## Introduction

Skin and hair follicle (HF) development and postnatal homeostasis are regulated by multifaceted programs of activation and silencing of gene expression. Through active research, substantial amount has been learnt about the essential regulatory networks that control mammalian skin development during embryonic and adult development and homeostasis by processes of self-renewal and differentiation of skin stem/progenitor cells [1, 2]. E74-like factor 5 (Elf5) is a member of the E26 transformation specific (ETS) family of transcription factors, which is expressed predominantly in epithelial cells and it has a wide spectrum of biological and physiological roles in epithelial development [3]. Elf5 has been shown to be a critical transcriptional regulator of cell fate in stem/progenitor cells by regulating target genes, such as, BMP, GATA3 and Notch signalling pathways [4–6], which are involved in cell proliferation and differentiation processes in development of epithelial tissues including the kidney [4] and mammary gland [7]. Furthermore, Elf5 is recognised as an important suppressor of EMT and inhibitor of metastasis in breast cancer [8]. Elf5 has also been implicated as a respiratory epithelial cell-specific risk gene for severe COVID-19 [9]. While these studies have firmly established Elf5 as a crucial regulator of cell fate determination of epithelial tissues, there has been very limited information of the expression and function of Elf5 in skin and HFs [10–12] and no knowledge if Elf5 is involved in the activities of epidermal and HF stem cells (SCs) and their progenies. Here we investigated Elf5 expression in skin and HFs during morphogenesis and postnatal development, as well as its regulation of keratinocyte proliferation and differentiation processes in skin.

## Material and methods

### Animals

Animal studies were performed in accordance with protocols approved by the UK Home Office Project License at Nottingham Trent University, UK. All methods are reported in accordance with ARRIVE guidelines. Wild type C57Bl/6 female mice were purchased from Charles River Laboratories. Skin samples were collected at defined stages during hair cycle-associated tissue remodelling [13] and snap frozen in liquid nitrogen. Animals were sacrificed by cervical dislocation and skin samples were taken during embryonic (E14.5-E18.5) and postnatal (PN) days 12–20.5. For depilation induced hair cycle, mice were anesthetized using ketamine and xylazine cocktail mixture (90–10 mg/kg, intraperitoneal injections) and kept in a heating pad during the depilation procedure. The backs of the mice were shaved using a hair clipper (Wahl) and depilated using warm wax. The warm wax was applied once to well stretched skin and removed at once against the direction of hair growth. After the depilation, to protect the skin of the depilated area, excess wax was removed using mineral oil (Merck, M8410). Mice were then allowed to recover on the heating pad and returned to their cages. For skin sample collection, mice were anesthetized with ketamine/xylazine cocktail mixture and then euthanized by cervical dislocation and samples taken at days 0–18 post depilation as described previously [11, 14] for immunofluorescent, and RT-qPCR analysis.

### Immunohistochemistry and immunocytochemistry

For immunohistochemical and immunocytochemistry analysis, 4% paraformaldehyde-fixed cells or cryostat sections (10μm) were blocked with 0.2% Triton-X-100/PBS, 5% fetal calf serum, 2% bovine serum albumin and 10% normal goat and/or donkey serum (Merck, UK) and incubated with primary antibodies against Elf5, Krt14, Krt15 or CD34

(S1 Table in S1 Data) overnight at 4°C. The following day, slides were incubated with the corresponding Alexa-Fluor-488, Alexa-Fluor-A555 or Alex-Fluro-Cy5 (S1 Table in S1 Data) secondary antibodies for 1 hour at room temperature. Incubation steps were interspersed with wash steps with 0.2% Triton-X-100/PBS. Sections were mounted with mounting medium with DAPI. Images were taken using Leica THUNDER imager 3D cell culture (Leica, Germany).

### Production of lentiviruses

HEK293T cells were cultured in DMEM (ThermoFisher, UK) supplemented with heat-inactivated 10% fetal bovine serum, 1% L-glutamine and 5% non-essential amino acids (ThermoFisher, UK) at 37°C, 5% CO_2_ as described previously [11]. In brief, for production of Elf5 overexpression, shElf5 (knockdown) or shRNA control lentiviruses: at 40% confluence HEK293T cells were co-transfected with mouse scrambled shRNA control pGFP-shRNA Vector (Cambridge Biosciences, UK), shRNA Elf5 Plasmid (shElf5) or Elf5 ORF clone plasmid (Elf5-OE, Cambridge Biosciences, UK) with packaging plasmid using Lenti-vpak packaging kit (Insight Biotechnology, UK) following manufacturer’s instructions. Cell culture medium containing viruses was collected 24 hours and 48 hours post-transfection, followed by precipitation of the viral particles using 80μg/ml polybrene (ThermoFisher, UK) and 80μg/ml chondroitin sulphate C (Merck, UK) for 18 hours at 4°C before centrifugation at 10,000xg. Titration of lentiviral particles was performed using qPCR lentivirus titration kit (NBS Biological, UK) as per manufacturer’s instruction.

### Fluorescent Activated Cell Sorting (FACS)

Skins from wild type C57Bl/6 mice were harvested between 7–9 weeks of age as described previously [11, 15–17]. In brief, mouse skin was dissected using Trypsin-EDTA (0.25%) and incubated for 2 hours overnight at 4°C. A scalpel and forceps were used to separate the epidermis from the dermis. The epidermis was subsequently minced and filtered using 40μm filter. The cells were centrifuged down at 300xg for 10mins and resuspended in 1ml of EMEM calcium-free medium (Lonza, UK). Cells were stained with Ly-6A/E (Sca-1) (ThermoFisher, UK), CD34 (RAM34) (ThermoFisher, UK) and CD49f (BD Pharmingen, USA) in 2% bovine serum albumin/PBS staining buffer rotating for 1 hour at 4°C. Cells were then stained with secondary antibody APC-streptavidin (Biolegend, USA) for 1 hour at 4°C. CD34^+^/CD49f^High^/Sca-1^−^; HF bulge SCs, CD34^−^/CD49f^high^/Sca-1^+^; epidermal keratinocyte SCs and CD34^−^/CD49f^Low^/Sca-1^−^; suprabasal keratinocytes were gated as described previously [18]. In brief, we initially set up primary gates based on DAPI to exclude dead cells and adjust the scatter plot (SSC-A vs FSC-A) to select for singlet events. Cells were gated based on CD34, CD49f (α6) and Sca-1 expression leading to three distinct cell populations as stated above. Cells were sorted using a MoFlo-XDP cell sorter (Beckman Coulter, UK) and data was analysed using Summit software (Beckman Coulter, UK). Sorted SCs were then used for subsequent colony forming assays and qRT-PCR analysis.

#### Colony forming assay

Swiss-3T3 cells were grown in DMEM media (ThermoFisher, UK) in 24-well plates at 37°C, 5% CO_2_. At 70% confluence, 3T3 cells were treated with 8μg/ml mitomycin C (ThermoFisher, UK) for 2 hours. Approximately, two-thousand HF bulge SCs or ten thousand epidermal SCs were seeded per 24-well plate and incubated for 48 hours with DMEM/F-12 media (3:1) supplemented with 10% chelated fetal bovine serum, 10ng/ml epidermal growth factor, 0.5μg/ml hydrocortisone, 10^-10^M cholera toxin, 5μg/ml insulin, 1.8^-4^M Adenine (Merck, UK), 100U/ml penicillin, 100μg/ml streptomycin and 0.3mM calcium. 48 hours post-seeding, SCs were transduced with Elf5-OE, shRNA Elf5 or shRNA control lentiviruses at a MOI of 20 (4.0 x 10^4^ viral titre/per ml) supplemented with 8μg/ml polybrene (ThermoFisher, UK) for 4 hours at 32°C, 5% CO_2_ [11, 19, 20]. Media was replaced every 48-hours and cultured for 7 days (HF SCs) or 10 days (epidermal SCs), respectively. GFP and Phase imaging was performed using IncuCyte S3 live cell analysis instrument (Sartorius, Germany).

### Rhodamine B staining

Post transduction of epidermal and HF SC colonies, SCs were incubated with Versene (ThermoFisher, UK) to remove the feeder cells. SCs were then wash in PBS and fixed with 4% PFA for 1 hour at room temperature. SCs were stained within the wells with Rhodamine B (ThermoFisher, UK, 1% Rhodamine in ddH_2_O) overnight at room temperature. Images were taken using Leica THUNDER imager 3D cell culture (Leica, Germany). Colonies were counted and diameter determined using the ImageJ macro, Cell Colony Edge as done previously [21].

### Cell culture and transfection

Cell culture and transfections of primary mouse epidermal keratinocytes (PMEKs) were prepared from wild type C57Bl/6 new-born mice at PN2-3 as described previously [22]. In brief, PMEKs were grown in EMEM calcium-free medium (Lonza, UK) supplemented with 0.05mM calcium (ThermoFisher, UK), 4% chelated heat-inactivated fetal bovine serum (ThermoFisher, UK), 0.4μg/ml hydrocortisone (Merck, UK), 5μg/ml insulin (Merck, UK), 10ng/ml epidermal growth factor (Merck, UK), 10^-10^M cholera toxin (Merck, UK), 2x10^-9^M T3 (Merck, UK), 100U/ml penicillin (ThermoFisher, UK), 100μg/ml streptomycin (ThermoFisher, UK), and 2mM L-glutamine (ThermoFisher, UK), at 33°C, 8% CO_2_ until 60–70% confluent.

PMEKs were transfected with Control plasmid (CloneTech, UK), Elf5 overexpression plasmid (Elf5-OE, Cambridge Biosciences, UK) or siRNA negative control (ThermoFisher, UK) and siElf5 SmartPool (knockdown; Dharmacon, UK,) using Lipofectamine 3000 (ThermoFisher, UK) as done previously [23]. Cells were harvested 48-hours post-transfection and used for further analyses.

For calcium-induced keratinocyte differentiation analysis, PMEKs were differentiated following transfection by media supplementation with 2.0mM calcium. PMEKs were cultured for an additional 48-hours post-transfection before collection for further analysis.

To assess the effects of loss of Elf5 on keratinocyte differentiation, we performed siRNA control and siElf5 knockdown in PMEKs for 48hrs, as described above, followed by calcium induced keratinocyte differentiation, as stated above, for additional 48-hours post transfection before cells were collected for further analyses.

### Reverse Transcription Quantitative Real-Time PCR (RT-qPCR)

Total RNA was isolated using the Zymo Direct-zol RNA kit (Cambridge Biosciences, UK). For gene expression analysis, 100ng of total RNA was converted into cDNA using the qPCRBIO cDNA Synthesis Kit system (PCR Biosystems, UK). Gene expression analysis was performed on QuantStudio5 Real Time PCR System (ThermoFisher, UK). Gene expression was analysed using qPCRBIO SyGreen mix (PCR Biosystems, UK) at the following conditions: 95°C for 2mins, followed by 40 cycles of denaturation (95°C for 5s), annealing and extension (30s at temperature experimentally determined for each primer pair). RT-qPCR primers (S2 Table in S1 Data) were designed using primer3 (https://primer3.ut.ee/) and further validated using UCSC genome browser (https://genome.ucsc.edu/) and NCBI primer blast (https://www.ncbi.nlm.nih.gov/tools/primer-blast/). Amplification differences between samples and controls were calculated based on the Ct (94^ΔΔ^Ct) method and normalized to mouse *Actin* gene (*Actb*). Data from triplicates were pooled, standard error of the mean (± SEM) was calculated, and statistical analysis was performed using unpaired student’s *t*-test and Two-way ANOVA test.

### Western blot

Whole cell protein lysates were extracted from cultured cells using RIPA lysis buffer (50mm Tris-HCl, 1% NP-40, 0.25% sodium deoxycholate, 150mm NaCl, and 1mm EDTA; pH 7.4) and cOmplete ULTRA Protease Inhibitor Cocktail (Merck, UK). Western blot was performed as described previously [23]. In brief, 10μg (micrograms) of protein were processed for western blot analysis, followed by membrane incubation with primary antibody overnight at 4°C. Horseradish peroxidase tagged IgG antibodies were used as secondary antibodies (1:3000, ThermoFisher, UK, S1 Table in S1 Data). Antibody binding was visualized with an enhanced chemiluminescence system (SuperSignal West Pico Kit, ThermoFisher, UK) on the FL1000 iBright GelDoc Imager (ThermoFisher, UK). Densiometric analysis was performed using Image J (https://imagej.net/ij/) (S3 Fig). Data shown as ratio relative to Gapdh (arbuitry units, a.u.) with standard error of the mean (± SEM), and statistical analysis was performed.

### Flow cytometry

PMEKs were cultures and transfected with Controls, pElf5-OE and/or siElf5 as described above. 48-hours post-transfection, cells were trypsinized, washed and fixed in 70% ethanol/PBS at -20°C for 30 mins. Fixed cells were incubated with RNase A (100μg/ml) for 30mins at 37°C. Cells were subsequently incubated with 20μg/ml Propidium iodide (ThermoFisher, UK) for 30mins at 4°C. The percentage of cells at distinct phases of the cell cycle was analysed by flow cytometry with a Beckman Coulter Gallios (Beckman Coulter, UK). For each sample, 10,000 events were collected and analysed on Beckman Coulter Kaluza Analysis Software (Beckman Coulter, UK) [11].

## Results and discussion

### Elf5 is dynamically expressed during skin and hair follicle development

**Fig 1:**
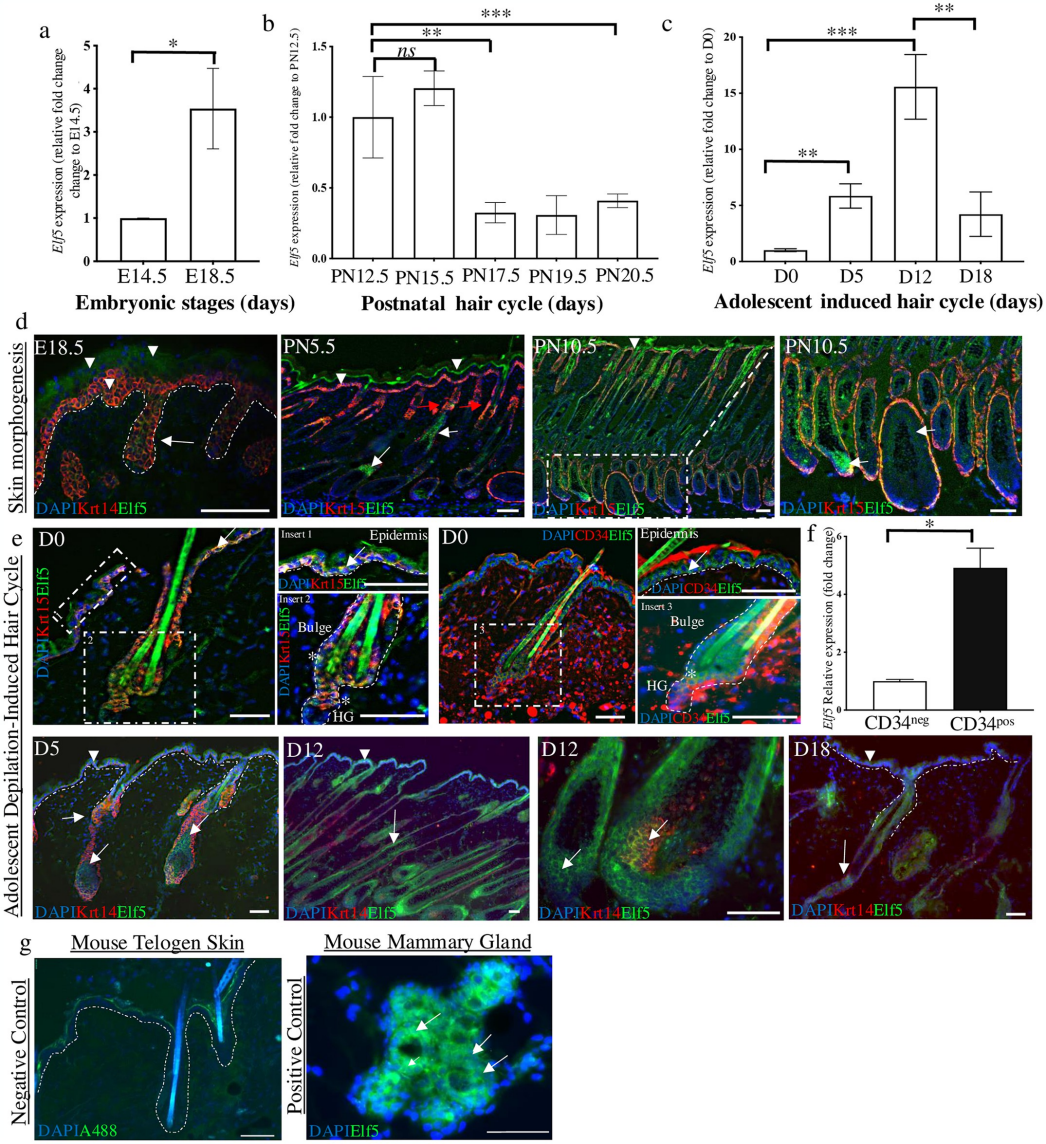
Elf5 is dynamically expressed in skin and hair follicles. (**a**) RT-qPCR analysis of *Elf5* expression in embryonic skin (E14.5 and E18.5); *Elf5* expression is upregulated in E18.5 skin compared to E14.5 skins. (**b**) *Elf5* levels in skin during the postnatal hair cycle: anagen-like stages (PN12-PN15.5), catagen (PN17.5), and telogen stages (PN19.5-PN20.5); *Elf5* expression is upregulated in anagen stages, with reduction in expression in catagen and telogen stages. (**c**) *Elf5* expression in adolescent depilation-induced hair cycle: telogen (day, D0), anagen (D5-D12), and catagen (D18). *Elf5* expression is elevated between telogen and anagen stages with a significant reduction in expression in catagen skin. Data are presented as mean ± SEM values from three independent experiments. *n* = 3 mice for each time point. (**d-e**) Immunohistochemstry staining of Elf5 during skin morphogenesis, Elf5 is detected in the basal and suprabasal layer of the epidermis (arrowheads) and developing hair bulb (arrows). In postnatal skin (PN5.5-PN10.5); Elf5 is expressed in the epidermis (basal and suprabasal, arrowheads), developing hair bulge and proliferative and differentiating keratinocytes of the developing hair bulb (arrows). During adolescent depilation-induced hair cycle; in telogen skin (D0), Elf5 expression is restricted to basal layer (Elf5^+^/K15^+^/CD34^−^, Insert 1, arrow) in the epidermis and within the HF bulge and hair germ stem cells (SCs) compartments (Elf5^+^/K15^+^/CD34^+^, Insert 2 and 3, asterisks). Progressive development of skin and HFs; Elf5 is expressed in epidermis (D5-D12, basal^Low^ and suprabasal^High^ expression, arrowhead). In HFs, Elf5 is consistently expressed in the highly proliferative and differentiating keratinocytes in the hair bulb matrix as well as in anagen HF bulge region (D5-D12, arrows). While, in catagen (D18), Elf5 expressed is weak in the epidermis (basal layer, arrowhead) and restricted to the regressing HF epithelium (arrow). Selected single channel images of staining have been provided in S1 Fig. (**f**) FACS isolation: C57Bl/6 wild-type mice aged 7–9 weeks were used to isolate HF bulge SCs. *Elf5* expression is elevated in CD34-postive HF bulge SCs compared with CD34-negative cells. Data are presented as mean ± SEM values from three independent experiments. *n* = 3 mice were used per experiment. (**g**) negative controls; using Alexa-fluor-488 (A488, green) and counter stained with DAPI (blue) in mouse telogen skin. No specific staining was observed. Positive control; mouse mammary gland showed both nuclear and cytoplasmic staining of Elf5 expression (green, arrows) in mammary gland alveoli. Images are representative microphotographs of staining. Cytokeratin 15 (Krt15). The broken lines demarcate the epidermal-dermal border. *p < 0.05; **p < 0.01 ***p < 0.001, *ns*, not significant; unpaired Student’s *t*-test. Scale bars: 50μm.

The expression of *Elf5* was examined during mouse embryonic (E14.5-E18.5), postnatal (PN12.5-PN20.5) development and adolescent depilated-induced hair cycle (day, D0-D18). RT-qPCR analysis of RNA isolated from full thickness skin revealed a dynamic change of *Elf5* expression throughout embryonic and postnatal skin stages; *Elf5* expression was elevated during late embryonic stages (E18.5) compared to E14.5 (*p value < 0.05, Fig 1a). In postnatal skin, *Elf5* expression was elevated in anagen stages (PN12.5-PN15.5) compared to lower expression observed in catagen (PN17.5) and telogen stages (PN19.5-PN20.5) (**p value < 0.01; ***p value < 0.001) (Fig 1b). A similar expression pattern was observed in skin at different time points of adolescent depilation-induced hair cycle, where elevated *Elf5* expression was detected in anagen (D5-D12) and catagen skin (D18) compared to telogen skin (D0, **p value < 0.01; ***p value < 0.001, Fig 1c).

Using immunohistochemistry, we observed Elf5 protein was expressed in the basal layer (Elf5^+^/Krt14^+^, Fig 1d) and in the differentiated suprabasal layer of the epidermis (Elf5^+^/Krt14^−^). While in the HFs, Elf5 expression was found in the pre-cortex and bulge regions (E18.5-PN10.5, Elf5^+^/Krt15^+^, Fig 1d). In adolescent depilation-induced hair cycle, Elf5 expression was restricted to the basal layer of the epidermis and in the HF bulge (Elf5^+^/K15^+^) and hair germ regions (Elf5^+^/K15^+^/CD34^+^) (D0, Fig 1e and S1 Fig). Furthermore, in advanced stages of anagen skin, Elf5 expression was found in the basal (Elf5^+^/K15^+^) and suprabasal layers of epidermis and in the HF bulge region. Of interest, Elf5 expression was observed in both the highly proliferative and differentiating cells of the anagen hair matrix (D5-D12). In catagen skin, weak Elf5 expression was restricted to the basal layer of epidermis and in the regressing epithelial strand of the HFs (D18, Fig 1e). Our data suggest that Elf5 expression found in the epidermis and HFs indicates the importance of Elf5 to balance and regulate proliferative potential of stem/progenitors, the transition of keratinocytes from proliferation to early differentiation and to also regulate the suprabasal expression of basal genes, thereby potentially acting as a switch between proliferation and differentiation of keratinocytes, a role similarly undertaken by Elf5 in mammary gland development [5, 24].

Of note, we detected cytoplasmic and nuclear Elf5 staining in epidermal and HF cells (Fig 1d and 1e and S1 Fig), which has also been observed in other closely related epithelial cells, such as in mammary (Fig 1g) and lung tissues [6, 9]. It is currently unknown if cytoplasmic Elf5 expression has any regulatory function in keratinocytes. However, previous studies have shown that cytoplasmic ELF5 staining can be a predictor of some breast cancer outcomes [25] and potentially has a functional significance in breast cancer [6]. Further analyses are required to better understand the importance of Elf5, and its role based on the spatio-temporal expression/localisation (nuclear *vs*. cytoplasmic) in particular in skin and HFs.

### Elf5 regulates epidermal and hair follicle stem cell colony forming abilities

To investigate if Elf5 is expressed in HF SCs, we FACS isolated CD34^+^ cells from the dorsal skin of 7-9-week-old wild type mice as done previously [11, 15–17]. CD34 is uniquely expressed in mouse HF bulge SCs and, therefore, has been used as a reliable molecular marker for HF SCs isolation [19, 26]. Using qRT-PCR, we found significantly higher levels of *Elf5* transcript (*p value < 0.05) in FACs isolated CD34^+^ compared to CD34^−^ cell populations (Fig 1f).

**Fig 2:**
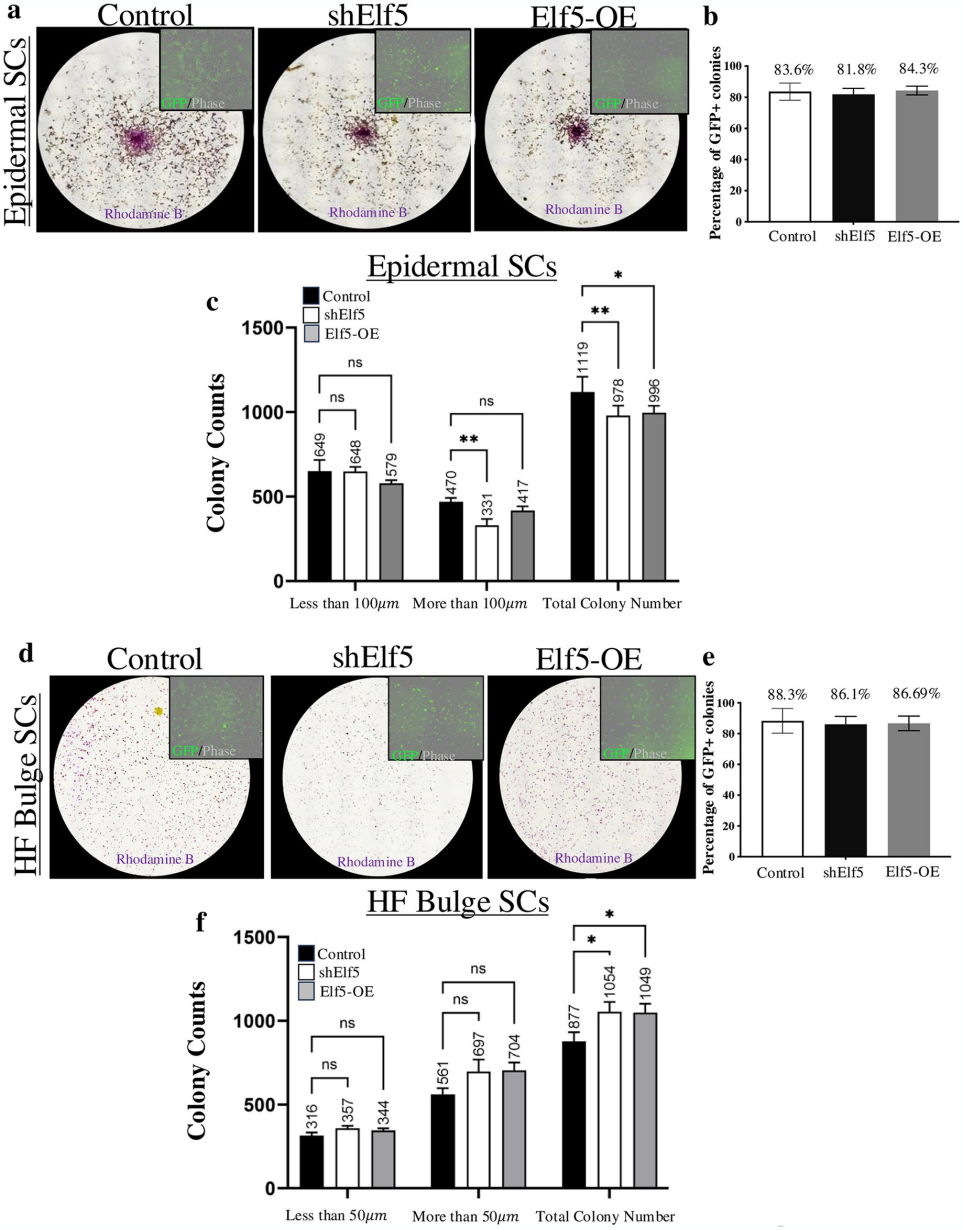
Elf5 can regulate colony forming abilities of epidermal and hair follicle stem cells. (**a-f**) Colony forming assay (CFA); representative images and quantification of the CFA assay of adult mice (7-9-week-old) epidermal and HF SCs grown, after transduction with lentiviral particles controls, shElf5 (knockdown) and Elf5 overexpression (OE), stained with 1% Rhodamine B. (**b,e**) Successful transduction of lentiviral particles was confirmed by the presence of >80% GFP^+^ cultured epidermal and HF bulge SC colonies (inserts). (**c,f**) Quantification of colonies formed after modulation of Elf5 functions in epidermal SCs leads to a significant reduction in size (>100μm, shElf5). In addition, significant decrease in total number of colonies being formed after loss and gain of Elf5 functions compared with control SCs. In HF SCs, a significant increase in the total number colonies being formed was observed after loss and gain of Elf5 activities compared to control SCs. Representative brightfield images of 2D colonies at day 7 (epidermal SCs) and day 10 (HF SCs) cultures, respectively, represented by overview images of individual wells of a 24-well plate at approximately 4× magnification. Data are presented as mean ± SEM values from three independent experiments. *p < 0.05; **p < 0.01, Two-way ANOVA test.

To investigate if Elf5 can regulate epidermal and HF SCs, we performed colony forming assays using FACs isolated cell populations as reported previously [11, 15]. FACs isolated epidermal [16, 27] and HF SC populations were validated by RT-qPCR (S2a and S2b Fig). We transduced both epidermal and HF SCs with control, shElf5 (knockdown) or Elf5 overexpression lenti-viruses and we confirmed successful transduction of lentiviral particles by the presence of GFP^+^ in cultured epidermal and HF-bulge SC colonies (>80% in both epidermal and HF SCs populations) (Fig 2b and 2e). Furthermore, using RT-qPCR, we validated Elf5 expression in these SC colonies after lentiviral treatments (S2c and S2d Fig). Interestingly, we observed opposing effects after loss-or-gain of Elf5 functions in epidermal SCs compared to HF SCs (Fig 2).

In epidermal SCs, loss-or-gain of Elf5 functions had no discernible impact on cell morphology as colonies were tightly packed and indistinguishable from controls (Fig 2a). However, we did observe that loss of Elf5 function leads to smaller number of colonies being formed compared to controls (>100μm; **p < 0.01, Fig 2c) as well as a significant reduction in total number of colonies being formed compared to controls (**p < 0.01, Fig 2c). Surprisingly, we observed that overexpression of Elf5 in epidermal SCs also leads to reduced total number of colonies being formed compared to controls (*p < 0.05; Fig 2c). In bulge HF SCs, loss-or-gain of Elf5 functions, colonies contained cells of small size and relatively undifferentiated morphology but were largely indistinguishable from controls (Fig 2d). Nonetheless, we did discover that loss and gain of Elf5 functions leads to significant (*p < 0.05) increase in total number of colonies being formed compared to controls but it had no significant impact on the size of the colonies (Fig 2d and 2f). The effects of loss or gain of Elf5 expression on both epidermal and HF SCs caused unexpected results. It is currently unknown how this occurs. However, one possible explanation for the seemingly surprising results is that Elf5 may act at multiple points along the SC lineages (in both epidermal and HF SCs), and a perturbation at one or more of these steps may directly impact the behaviour of SCs. Consistent with this possibility, previous studies have also shown that after modulation of Elf5 functions in other epithelial cells/tissues can lead to increased pool of mammary SCs and stem/progenitor cell populations in lung epithelium, *in vivo* [3, 8]. Furthermore, loss and gain of Elf5 functions result in depletion of trophoblast SCs, *in vitro* and *in vivo* [28]. Collectively, these data suggest that Elf5 may act as rheostat controlling and balancing cell fate by regulating transcriptional networks governing self-renewal, quiescence and lineage determination in epidermal and HF SCs. However, further analyses are required to determine its mechanistic role in these cell SC populations.

### Elf5 inhibits proliferation and promotes keratinocyte differentiation

**Fig 3:**
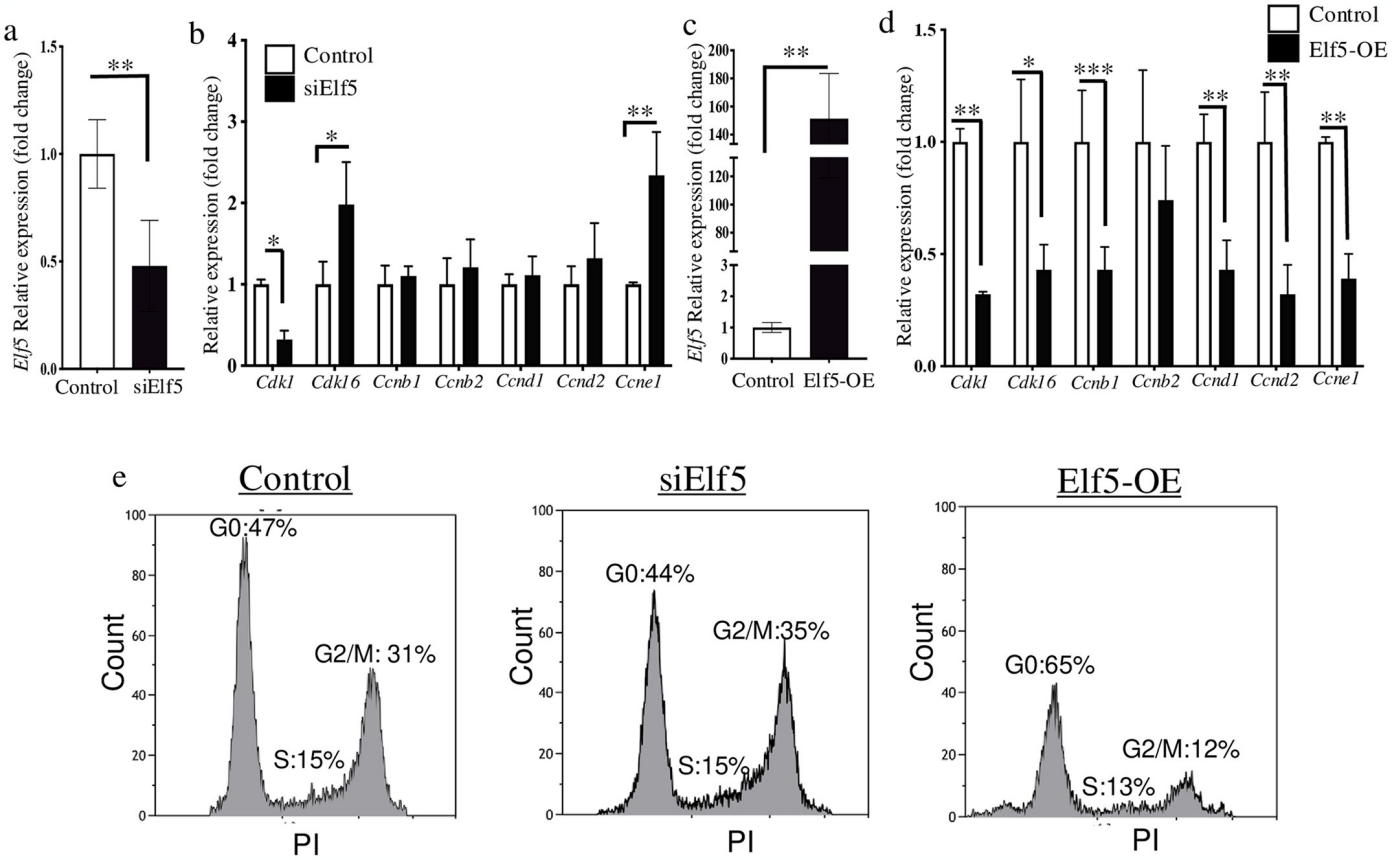
Elf5 can inhibit epidermal keratinocyte proliferation. (**a-d**) RT-qPCR analysis of Elf5 and cell cycle gene markers in PMEKs showed an increase and/or decrease in the expression of genes analysed after modulation of Elf5 activities, respectively. Data are presented as mean ± SEM values from three independent experiments. (**e**) Flow cytometric analysis by PI in PMEKs showed accumulation in G0-phase and, subsequently, a reduction of cells entering the S and G2/M phases of the cell cycle after Elf5 overexpression (OE). Limited effects were observed on PMEKs after siElf5 treatment compared to controls. The graphs shown are from a single representative experiment. Percentages are presented as mean values from three independent experiments. PI, propidium iodide. PMEKs, primary mouse epidermal keratinocytes. *p < 0.05; **p < 0.01; ***p < 0.001; unpaired Student’s *t*-test.

To explore the potential role of Elf5 in epidermal keratinocytes, we functionally assessed Elf5 impact on keratinocytes proliferation and differentiation processes. Loss of Elf5 functions (Fig 3a) in epidermal keratinocytes resulted in significantly increased expression on proliferative genes (*Cdk1*, *Cdk16* and *Ccne1*,” *p<0.05; **p<0.01, Fig 3b). In addition, a significant decrease in transcripts levels of *Cdk1*, *Cdk16*, *Ccnb1*, *Ccnd1*, *Ccnd2* and *Ccne1* in epidermal keratinocytes was caused by Elf5 overexpression (*p < 0.05; **p < 0.01; ***p < 0.001, Fig 3c and 3d). Subsequently, cell cycle analysis by flow cytometry revealed limited impact on primary keratinocytes after loss of Elf5 on cell cycle progression (Fig 3e). While Elf5 overexpression leads to G0 arrest compared to controls cells (Fig 3e). This data suggest that Elf5 can potentially inhibit epidermal keratinocyte proliferation and promote their differentiate. Importantly, both *Cdk16* and *Ccne1*, which are significantly up-and downregulated after modulating Elf5 expression are critical regulators of cell cycle progression, and their deregulation can lead to disease or cancer formation [29]. Our observations are consistent with previous studies showing that loss of Elf5 has limited effects on trophoblast proliferation [30, 31]. While overexpression of Elf5 promotes progenitor cell differentiation in mammary gland [32]. The processes of cellular proliferation and differentiation are tightly linked to the maintenance of epidermal homeostasis. This balance is maintained by several crucial transcriptional (e.g. p63) and signalling pathways (e.g. TGFβ) in skin [33, 34]. Thereby, our data suggest that Elf5 can act as a switch between proliferation and differentiation of keratinocytes, by balancing and regulating the transition of keratinocytes from proliferation to early differentiation, which if deregulated could lead to disease states in skin.

**Fig 4:**
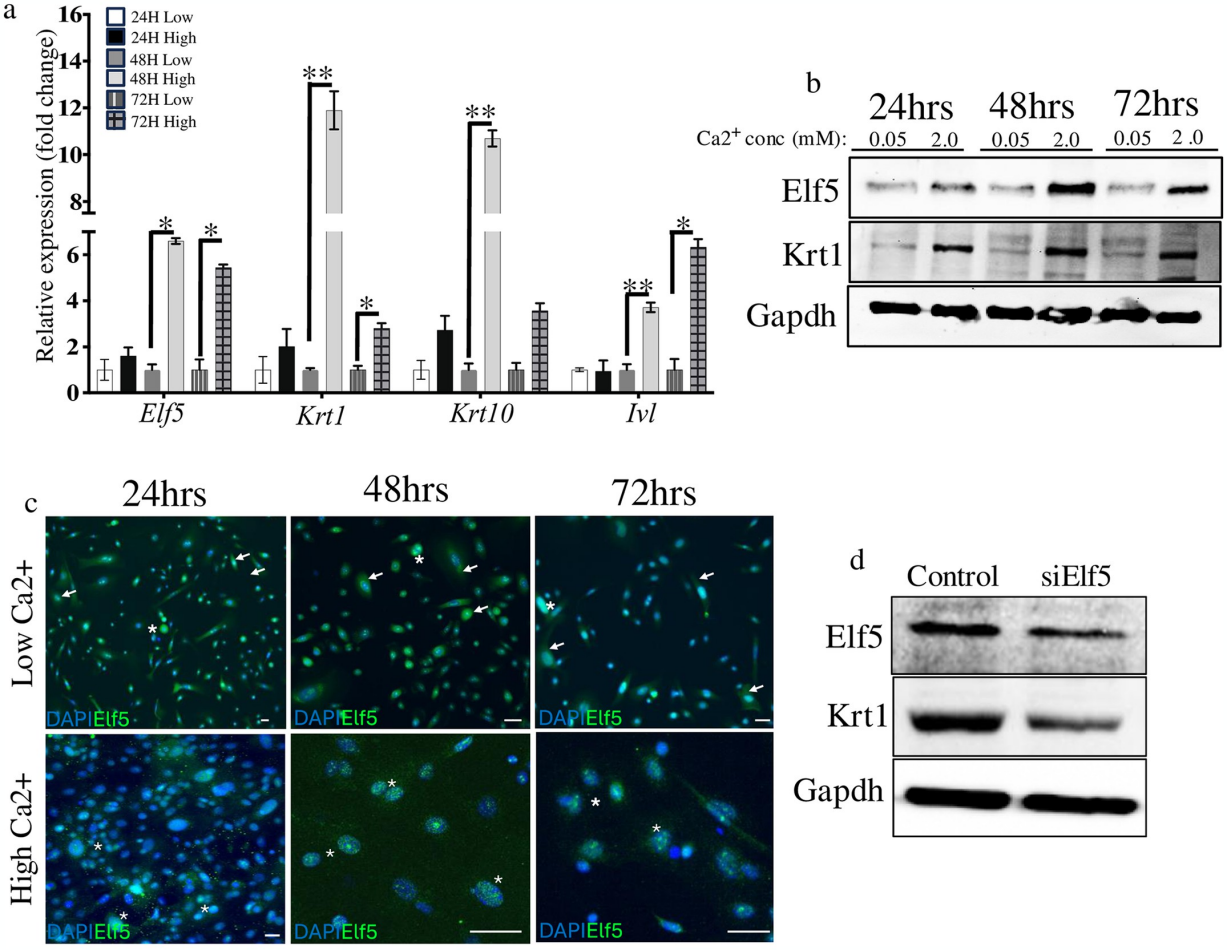
Elf5 can promote epidermal keratinocyte differentiation. (**a**) RT-qPCR analysis during calcium (Ca2^+^)-induced (low: 0.05mM or high: 2.0mM) keratinocyte differentiation in PMEKs, showing an increase in the expression of keratinocyte differentiation-associated markers cytokeratin 1 (*Krt1*), *Krt10*, and *Involucrin* (*Ivl*) and *Elf5*, respectively. Data are presented as mean ± SEM values from three independent experiments. (**b**) Western blot analysis of Elf5 and cytokeratin 1 (Krt1) during calcium-induced keratinocyte differentiation in PMEKs; consistent with RT-qPCR, Elf5 and Krt1 protein levels are elevated in calcium-induced PMEKs overtime. (c) Immunocytochemistry analysis: prominent nuclear Elf5 staining (green) is observed in PMEKs during calcium-induced keratinocyte differentiation (high Ca2^+^) over time compared with low Ca2^+^ treated cells. Cells were counterstained with DAPI (blue). The data shown are from a single representative experiment out of three experimental repeats. (d) While loss of Elf5 function leads to inability of keratinocytes to differentiate properly as observed by reduced expression of Krt1 protein levels in differentiated PMEKs. Data shown are from a single representative experiment of three experimental repeats. Densiometric analysis can be found in S3 Fig. *p < 0.05; **p < 0.01; unpaired Student’s *t*-test. PMEKs, primary mouse epidermal keratinocytes. Scale bars: 20μm.

Next, we analysed Elf5 potential role during epidermal keratinocyte differentiation. We observed elevated levels of Elf5 transcript and protein in keratinocytes during their calcium-induced differentiation, *in vitro*, which was accompanied with the induction of the differentiated-associated genes, *Krt1*, *Krt10* and *Involucrin* (*Inv*) (Fig 4a and 4b and S3a Fig). Consistent with these observations, immunofluorescence analysis confirmed that Elf5 expression increases and is more localised in the nucleus of PMEKs overtime during calcium induced keratinocyte differentiation, *in vitro* (Fig 4c).

Loss of Elf5 in keratinocytes during calcium-induced differentiation inhibited the ability of keratinocytes to differentiate properly as was confirmed by reduce Krt1 expression (Fig 4d and S3b Fig). Our data are consistent with previous studies showing that modulation of Elf5 expression has a direct impact on epithelial differentiation processes [3, 32]. Our data suggest that in the epidermis, Elf5 may function, in part, by restricting the proliferative potential of stem/progenitors as they transitioned from basal to suprabasal layers. This regulation may explain how proliferating basal epidermal keratinocytes commit to differentiation and how the epidermis is protected from cell growth abnormalities and disease. Although the mechanisms underlying the balance between proliferation and differentiation processes in skin are complex, our data suggest that Elf5 is likely to be important regulator of transcriptional controls regulating proliferation, differentiation, and SC survival, which requires further investigation.

As Elf5 is expressed in the epidermal basal and HF SC cell populations, we were interested to determine if Elf5 had any regulator impact on master transcriptional regulator p63. Of interest, a previous study has shown Elf5 to regulate p63 expression [3]. We therefore sought to determine if regulation of p63 by Elf5 was potentially conserved in skin. Previous studies have shown that p63 knockout (^−/−^) mice fail to form stratified epithelium and failed to maintain stem/progenitor cell population in skin and HFs [35–38]. Similarly, Elf5 knockout (^−/−^) mice inhibits stem/progenitor function of the mammary epithelium and blocked mammary basal cell differentiation [5]. We therefore analysed DeltaNp63α, which is the predominant isoform expressed in the epidermis [38] and we observed that overexpression of Elf5 in keratinocytes induced *DeltaNp63α* expression, while loss of Elf5 leads to a decrease in *DeltaNp63α* expression (S4 Fig). Our data is consistent with a previous study [3] and suggests a potential important and highly conserved mechanism of regulation of p63 by Elf5 in keratinocytes. Our data also suggests that Elf5 likely triggers a different set of effectors that determine whether a cell should proliferate or begin to differentiate in part by regulating p63 expression. Determining the specific events or signalling cascades involved remain an outstanding question and requires additional studies.

Subsequently, as our data suggest a potential Elf5 regulation of p63 in keratinocytes, this opens up the possibility of use of Elf5 as a therapeutic target for p63 pathway specific skin abnormalities. As p63 is a key regulator of epidermal lineage commitment, epidermal differentiation and cell adhesion [39], the use of Elf5 to modulate p63 expression may provide some benefits to patients. However, additional studies are required to determine its application.

## Conclusion

In summary, our data reveal Elf5 as a potential key determinant that controls the activity of epidermal and HF SCs and of keratinocytes proliferation and differentiation processes in the developing and postnatal skin and HFs. As Elf5 has an important regulatory role in stem/progenitor cell activities in many other epithelial tissues during development and regeneration [4, 5] and loss of Elf5 can lead to defects in epithelial development [31] and tumourigenesis [40], therefore, these data provide an important foundation for further analyses of the role of Elf5 in many areas of research, including stem cell and cancer biology, regenerative medicine and ageing.

## Supporting information

S1 Fig Single channel images of immunofluorescent staining. We have provided a selection of single channel images of immunofluorescent images to demonstrate the localised expression of Elf5 in telogen skin and hair follicles (from Fig 1e, telogen stage, day 0 of adolescent depilation-induced hair cycle). Staining consists of Elf5 (green), Cytokeratin 15 (Krt15, red) and counterstained with DAPI (blue). Both nuclear and cytoplasmic Elf5 expression can be observed in the epidermis and within the hair follicle stem cells compartments (arrowhead and arrows, Panels b,c,d,f). Negative controls have been provided in Fig 1g. The broken lines demarcate the epidermal-dermal border. Scale bars: 50μm (Panels a-b,d-e) and 10μm (Panels c,f).
(PDF)

S2 Fig RT-qPCR validation of FACs isolated epidermal and hair follicle bugle stem cells. (**a**) RT-qPCR analysis of FACs isolated hair follicle (HF) bulge stem cells (SCs); elevated expression of *Lgr5*, *Lhx2* and *Cytokeratin 15* (*Krt15*) in CD34-postive SCs compared to CD34-negative confirming appropriate isolation of HF SC populations. (**b**) In addition, RT-qPCR analysis of FACs isolated epidermal SCs; elevated expression of *Itga6*, *Krt14* and *Krt5* in CD34^−^/α6^High^/Sca1^+^ epidermal SCs compared to CD34^−^/α6^Low^/Sca1^−^ suprabasal cell confirming appropriate isolation of epidermal SC populations. (**c-d**) Transduction efficiency of FACs isolated epidermal and HF SCs with controls, shElf5 (knockdown) or Elf5 overexpression (OE) lentiviruses, were validated by qRT-PCR analysis confirming successful knockdown and overexpression of *Elf5* in both epidermal and HF SCs post transduction compared to controls SCs, respectively. Data are presented as mean ± SEM values from three independent experiments. *p < 0.05; **p < 0.01; ***p < 0.001; unpaired Student’s *t*-test (**a-b**) and Two-Way ANOVA test (**c-d**).
(PDF)

S3 Fig Densiometric analysis. (**a-b**) Densiometric analysis was performed using Image J (https://imagej.net/ij/). Data shown as ratio relative to Gapdh (arbuitry units, a.u.) with standard error of the mean (± SEM) from three independent experiments. *p < 0.05; **p < 0.01; ***p < 0.001; unpaired Student’s *t*-test. Cytokeratin 1 (Krt1). L: low calcium (0.05mM), H: high calcium (2.0mM).
(PDF)

S4 Fig DeltaNp63α expression is modulated in epidermal keratinocytes after loss-or-gain of Elf5 functions. (**a**) RT-qPCR analysis of *DeltaNp63α* in primary mouse epidermal keratinocytes showed an increase and/or decrease in expression after modulation of Elf5 activities, respectively. Data are presented as mean ± SEM values from three independent experiments. **p < 0.01; unpaired Student’s *t*-test.
(PDF)

S1 Data S1 and S2 Tables: List of antibodies and mouse RT-qPCR primers. (XLSX)
