# Supplementary figures and images for: The involvement of Elf5 in regulating keratinocyte proliferation and differentiation processes in skin
Source: PLoS One. 2025 Jan 3;20(1):e0316134. doi: 10.1371/journal.pone.0316134 (PMC11698348; doi:10.1371/journal.pone.0316134)

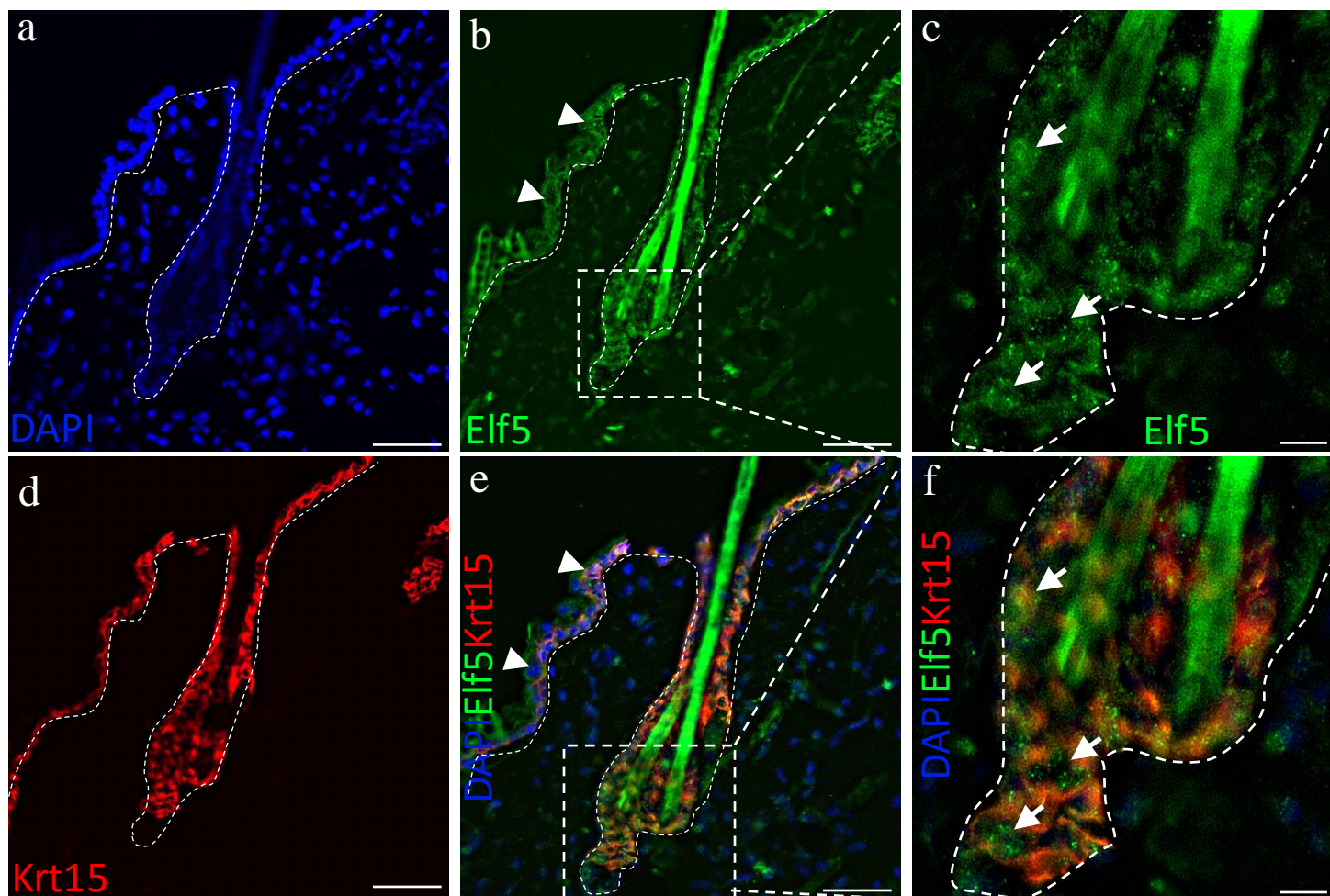

**Supplemental Figure S1**

Supplement: S1 Fig — We have provided a selection of single channel images of immunofluorescent images to demonstrate the localised expression of Elf5 in telogen skin and hair follicles (from Fig 1e, telogen stage, day 0 of adolescent depilation-induced hair cycle). Staining consists of Elf5 (green), Cytokeratin 15 (Krt15, red) and counterstained with DAPI (blue). Both nuclear and cytoplasmic Elf5 expression can be observed in the epidermis and within the hair follicle stem cells compartments (arrowhead and arrows, Panels b,c,d,f). Negative controls have been provided in Fig 1g. The broken lines demarcate the epidermal-dermal border. Scale bars: 50μm (Panels a-b,d-e) and 10μm (Panels c,f). (PDF) [file pone.0316134.s001.pdf]

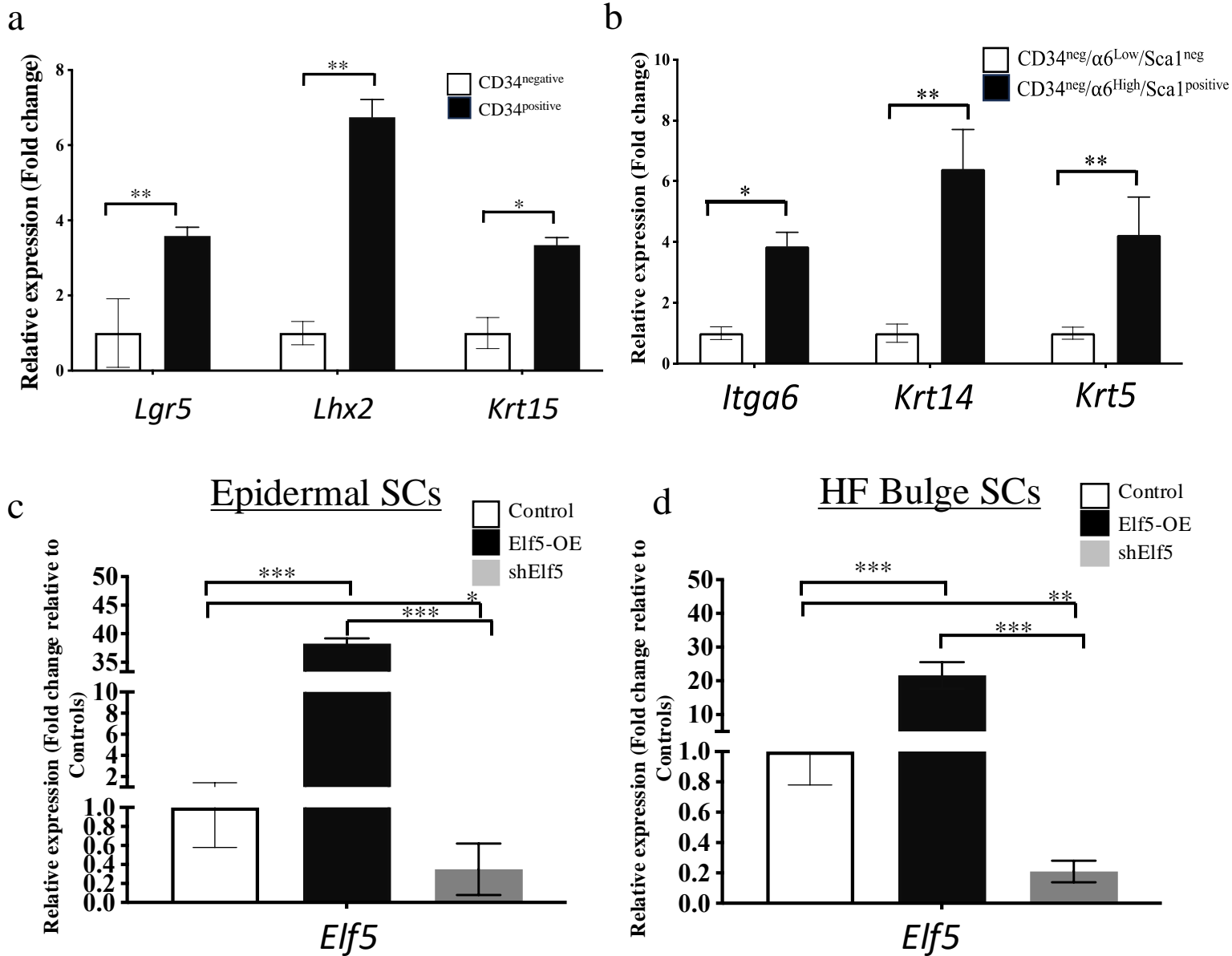

**Supplemental Figure S2**

Supplement: S2 Fig — (a) RT-qPCR analysis of FACs isolated hair follicle (HF) bulge stem cells (SCs); elevated expression of Lgr5, Lhx2 and Cytokeratin 15 (Krt15) in CD34-postive SCs compared to CD34-negative confirming appropriate isolation of HF SC populations. (b) In addition, RT-qPCR analysis of FACs isolated epidermal SCs; elevated expression of Itga6, Krt14 and Krt5 in CD34−/α6High/Sca1+ epidermal SCs compared to CD34−/α6Low/Sca1− suprabasal cell confirming appropriate isolation of epidermal SC populations. (c-d) Transduction efficiency of FACs isolated epidermal and HF SCs with controls, shElf5 (knockdown) or Elf5 overexpression (OE) lentiviruses, were validated by qRT-PCR analysis confirming successful knockdown and overexpression of Elf5 in both epidermal and HF SCs post transduction compared to controls SCs, respectively. Data are presented as mean ± SEM values from three independent experiments. *p < 0.05; **p < 0.01; ***p < 0.001; unpaired Student’s t-test (a-b) and Two-Way ANOVA test (c-d). (PDF) [file pone.0316134.s002.pdf]

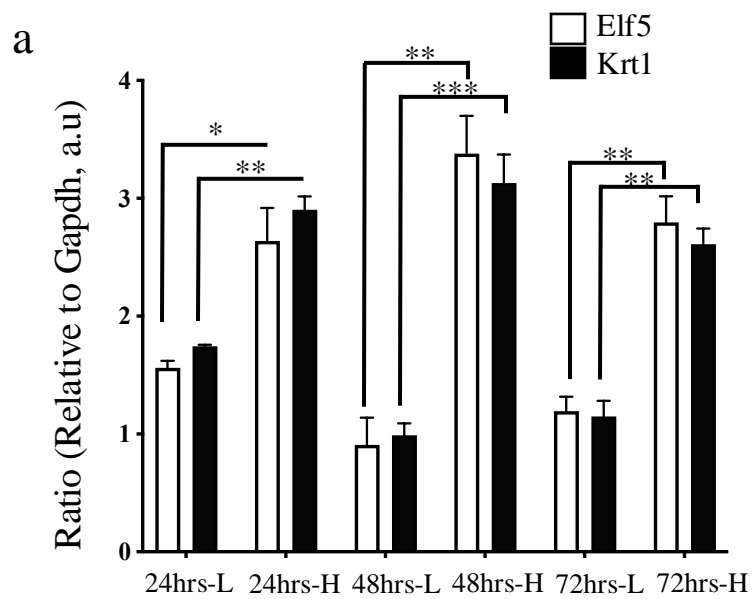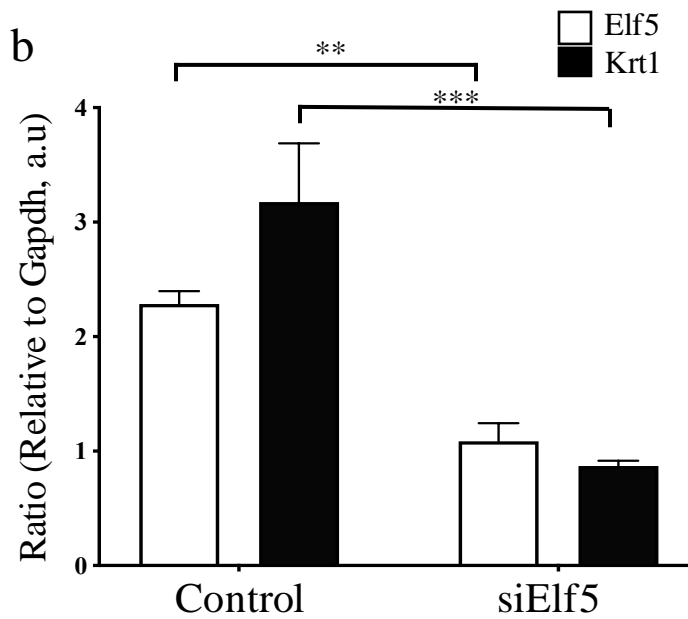

**Supplemental Figure S3**

Supplement: S3 Fig — (a-b) Densiometric analysis was performed using Image J (https://imagej.net/ij/). Data shown as ratio relative to Gapdh (arbuitry units, a.u.) with standard error of the mean (± SEM) from three independent experiments. *p < 0.05; **p < 0.01; ***p < 0.001; unpaired Student’s t-test. Cytokeratin 1 (Krt1). L: low calcium (0.05mM), H: high calcium (2.0mM). (PDF) [file pone.0316134.s003.pdf]

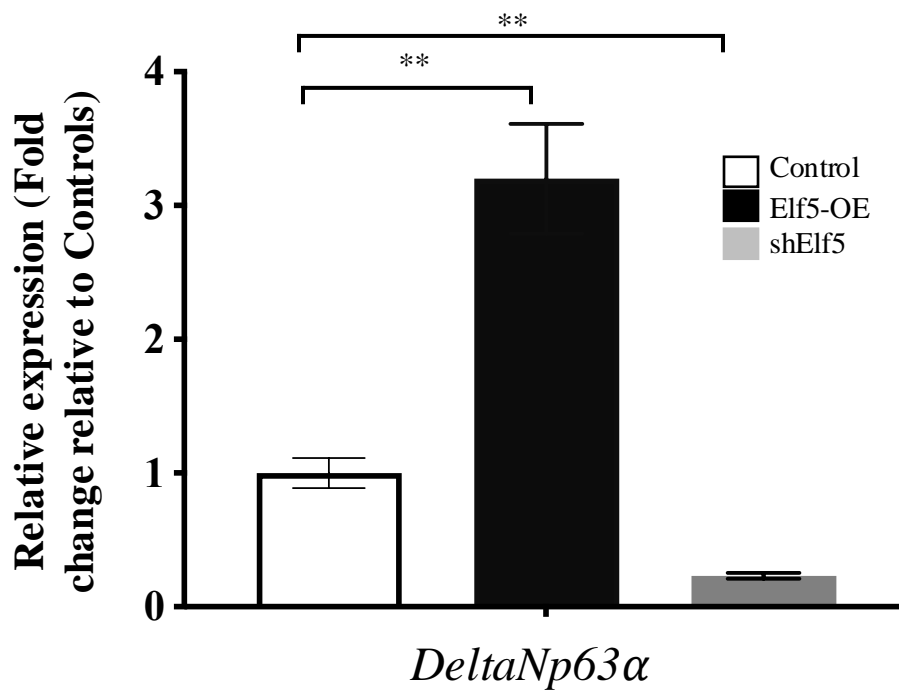

**Supplemental Figure S4**

Supplement: S4 Fig — (a) RT-qPCR analysis of DeltaNp63α in primary mouse epidermal keratinocytes showed an increase and/or decrease in expression after modulation of Elf5 activities, respectively. Data are presented as mean ± SEM values from three independent experiments. **p < 0.01; unpaired Student’s t-test. (PDF) [file pone.0316134.s004.pdf]
